# Temporal Activity in Particular Segments and Transitions in The Olympic Triathlon

**DOI:** 10.2478/hukin-2013-0009

**Published:** 2013-03-28

**Authors:** Roberto Cejuela¹, Antonio Cala, José A. Pérez-Turpin¹, José G. Villa, Juan M. Cortell¹, Juan J. Chinchilla¹

**Affiliations:** 1 Departmental Section of Physical Education and Sports, University of Alicante (Spain).; 2 High Performance Sport New Zealand, New Zealand.; 3 Department of Physical Education and Sports, University of León (Spain).

**Keywords:** cycle-run transition, swim-bike transition, triathlon

## Abstract

The Olympic Triathlon is a combined endurance sport. It includes back-to-back swimming, cycling, running and the transition between events (T1 & T2). The aim of the current study was to analyse the possible relationship between the Lost Time T1 & T2 and overall performance. The results showed that the percentages of total time corresponding to each part of the race were: 16.2% for swimming, 0.74% for the swimming-cycling transition (T1), 53.07% for cycling, 0.47% for the cycling-running transition (T2) and 29.5% for running. The correlations between each part of the race and the final placing were: r=0.36 for swimming, r=0.25 for T1, r=0.62 for the cycling, r=0.33 for T2, and r=0.83 for the running. Also, values of r=0.34 & r=0.43 were obtained for Lost Time T1 and Lost Time T2, respectively. In conclusion, losing less time during T2 has been demonstrated to be related to obtaining a better final result.

## Introduction

The Olympic triathlon involves a 1.5 km swim, a 40 km cycle and a 10 km run completed under “draft-legal” conditions (Bentley et al., 2002). In order to be selected for the Olympics, the athletes must obtain an Olympic qualification ranking, via a competition system where points are obtained according to the placing in those races. The most common events used for this ranking are the ITU World Cups (Bentley et al., 2002).

Numerous studies have investigated the physiology of triathlon in laboratory-based conditions (Sleiver et al., 1996; Bentley et al., 2002, 2007a, 2007b; Hue et al., 1998, 2002; Millet et al., 2000, 2002). Currently, experiments are carried out to describe the physiological requirements of competition where external performance factors are considered (Cejuela et al., 2007; Vleck et al., 2008; Cala et al., 2009; Le Meur et al, 2011). Triathlon represents an interesting model to examine differences in performance as the time differences can be analyzed for three different endurance disciplines.

Since 1981, elite male and female triathletes have improved their performances at the Hawaii Ironman Triathlon (Lepers, 2008). However, there are no studies describing the evolution of performance in Olympic-distance triathlon over the years.

Several studies have indicated a progressive reduction in speed, power output and heart rate during the event. The Olympic-distance triathlon requires a higher aerobic and anaerobic demands than constant-workload cycling exercises previously analyzed in laboratory conditions (i.e., time trial) or Ironman triathlons (Bernart et al., 2009; Le Meur et al., 2009).

Other studies analyzed the pacing strategies of the triathletes during the running segment, showed that the first kilometers were run faster. This higher running speed may be due to the high pacing at what T2 (bike-run transition) is performed (Vleck et al., 2008; Le Meur et al., 2011).

The “Lost Time” for the swim-cycle (T1) and cycle-run (T2) transitions corresponds to the time difference between each competitor and the tri-athlete that started the bike (T1) or the run (T2) first (Cejuela et al., 2008). To the best of our knowledge, no study has analysed the possible relationship between the Lost Time T1 and T2 and the overall performance. Our hypothesis is that making the T2 faster, will improve the overall performance significantly.

Therefore, the aim of the present study was to analyse the temporal activity of the different segments and transitions of the triathlon over the years in international competitions (nine top-level Olympic-distance events) and relate it to the final performance in these competitions.

## Material and Methods

Nine top-level men triathlon competitions held from 2000 to 2008 were studied: 6 World Championships (2000, 2001, 2004, 2006, 2007 and 2008) and 3 Olympic Games (2000, 2004 and 2008). The total number of participants was 537 (n=537), with 59.67±11.08 (mean±SD) participants per competition. All the tri-athletes who finished the race were considered for the analysis. We discarded the partial results of competitors who were disqualified or retired. All the participants gave their informed written consent to take part in this study that was conducted according to the Declaration of Helsinki. The Ethics and Research Committee of the Alicante University approved the study.

We gathered the data for all events in collaboration with the International Triathlon Union (ITU). In order to gather the times for all competitions we used the “ChampionChip®” microchip timing system. All athletes wore the chip on their left ankles during the races. When they crossed the reading mats, the partial times for each segment, transition and total competition times were recorded. These mats were placed at the start, entrance/exit to/from the transition area and at the finish line. The data at the 2002, 2003 and 2005 World Championships were not analysed due to the fact that the timing system did not record the time taken to carry out the transitions separately (T1 & T2) but included them into the cycling time.

### Determination of lost time in T1 and T2

Lost time in transitions T1 and T2 is the time lag between the first tri-athlete who starts cycling or running leaving the transition area, and the rest of the triathletes who arrived at the transition area in the same swimming or cycling pack.

This time depends on two factors. Firstly, the tri-athlete’s position in the swimming or cycling pack when entering the transition area. The lower the rank is, the longer is the time lost during transition and vice versa. The higher the rank is, the less time is lost. Secondly, the time taken by the triathlete to carry out the specific actions required in the transition area, as changing equipment and crossing the designated area. This time is only valid as a reference for the swimming or cycling pack in which each triathlete reaches the transition area. It cannot be compared with other groups getting into the transition areas at different times.

The time lost in T1 and T2 can be calculated by filming and analysing the videos of each entrance and exit from the transition area (Cejuela et al., 2008) or by mathematical calculations based on partial times.

Lost time in T1 is calculated by the difference (in seconds) between the best partial accumulated time (at the end of T1) and the partial accumulated time of each tri-athlete belonging to the same swimming pack. The criteria used to decide whether two tri-athletes belong to the same pack is when the difference between them at the end of the swimming segment does not exceed 5 seconds.

### Lost Time T1=Best partial accumulated time – accumulated time of each triathlete in the same swimming pack

#### Accumulated time=Time for the swimming segment + time for the swimming-cycling transition (T1)

Lost time in T2 is calculated by the difference (in seconds) between the best partial accumulated time (at the end of T2) and the partial accumulated time of each tri-athlete belonging to the same cycling pack. As in T1, the criteria used to decide whether two tri-athletes belong to the same pack is when the difference between them at the end of the cycling segment does not exceed 5 seconds.

#### Lost Time T2=Best partial accumulated time – accumulated time of each triathlete in the same cycling pack

#### Accumulated time=Time for the swimming segment+Time for transition T1+Time for the cycling segment+Time for transition T2

The reason to set five seconds as the bench mark is based on results found in the literature. Hydrodynamic resistance calculations have shown that the ideal distance to draft behind another tri-athlete has not been exactly determined. However, it has been demonstrated that swimming more than five seconds behind the preceding tri-athlete does not provide any advantage over swimming alone (Chatard et al., 1998; Bentley et al., 2007).

Similar studies in cycling have shown that riding with practically inexistent separations between wheels can lead into 44% reduction in aerodynamic resistance, and up to 27% with a separation of two metres (McCole et al., 1990; Lucía et al., 2001; Faria et al., 2005). This is the main reason why five seconds have also been used as the bench mark in the cycling segment to consider whether two tri-athletes belong to the same pack.

### Data analysis

Standard statistical methods were used to calculate mean, SD, and percentages. Time distribution was assessed via a general linear model with repeated-measures analysis of variance (ANOVA) to compare swimming, cycling, and running. Additionally, a Levene test for homogeneity of variances was completed on each dependent variable during the ANOVA, and, in each case, homogeneity of variance was found. Post hoc comparisons were completed using a Tukey HSD least significant difference. The T test was used to determine differences in T1 and T2. Pearson correlation coefficients were used to determine the relationships between each segment, transition and lost time T1 & T2 and the sport achievement. For all tests, the significance level was set at p<0.05 and p<0.001. The analyses were done using SPSS 15.0 (SPSS Inc. Chicago, IL). The coefficient of variation was used as a measure of intra-individual variation in time distribution for each competition and total time spent during competitions and was calculated as the standard deviation of the difference between repeated measurements divided by the mean and multiplied by 100 (Atkinson and Nevill, 1998).

## Results

Table 1 shows the mean (±SD) time spent for each segment, transition and total time for all the competitions analysed. The mean total time spent by tri-athletes to finish the races was 1 hour, 52 minutes and 5 seconds ± 4 min. The longest segment was cycling, followed by running and swimming. T1 lasts longer than T2. T1 was the part of the race with the greatest variability.

Table 2 compares the average times of each part of the race of all the participants (swim: 18min 19s ± 25s, 6.89% CV (coefficient of variation); T1: 42s ± 16s, 33.83% CV; bike: 59min 9s ± 3min 41s, 6.73% CV; T2: 19s ± 7s, 27.17% CV; run: 33min 30s ± 44s, 5.68% CV; total time: 1h 52min 5s ± 4min, 4.58% CV) and the top 10 (swim: 18min 18s ± 25s, 2.47% CV; T1: 44s ± 15s, 37.12% CV; bike: 58min 48s ± 3min 27s, 5.68% CV; T2: 26s ± 7s, 31.4% CV; run: 31min 31s ± 43s, 2.64% CV; total time: 1h 49min 32s ± 3min 53s, 3.38% CV) with the values of the winners (swim: 18min 9s ± 25s, 2.26% CV; T1: 39s ± 15s, 38.92% CV; bike: 57min 56s ± 3min 20s, 5.76% CV; T2: 26s ± 9s, 35.56% CV; run: 31min 3s ± 51s, 2.71% CV; total time: 1h 48min 13s ± 3min 44s, 3.43% CV). Significant differences (p<0.05) for the total time and for the running section were found (0.01).

There are significant differences between the mean time spent on T1 and T2 for all participants (43.74 ± 14.79s T1, T2: 28.65 ± 7.78s), the top 10 (40.07 ± 14.88s T1, T2 26.59 ± 8.35s) and winners (38.89 ± 15.14s T1, T2: 26 ± 9.25s). Therefore, no difference between groups was observed.

Table 3 shows the percentage of the total time (%) relative to each segment and transition. Cycling presents a higher value (52.73 ± 1.47%) than running (29.9 ± 0.72%) and swimming (16.35 ± 0.62%), while the transitions only account for 1.3 ± 0.33% of the total duration of the competition.

The percentages of the winners are very similar to the values obtained for the other competitors. Only the running segment showed significant differences (28.70 ± 0.58 winners; 29.14 ± 0.7 top 10; 29.90 ± 0.72 total) between the groups.

**Figure 1:**
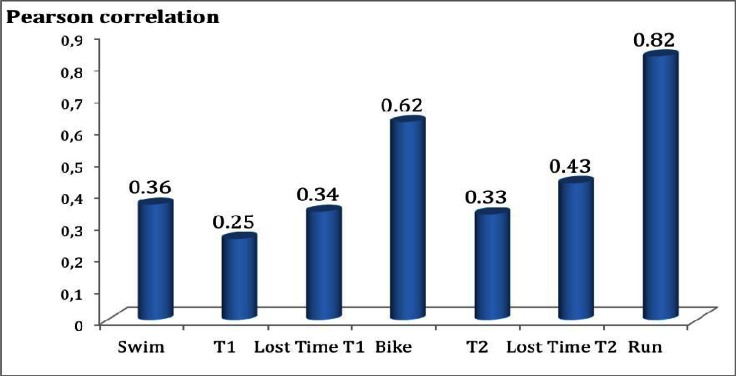
The mean of the Pearson correlation coefficients obtained (p<0.001) between each segment, transition and lost time T1 & T2 and the final classification of a triathlon competition

In order to see whether the time distribution within the race had any relationship with the overall performance, correlations between each part of the race (including lost time in T1 & T2) and the final classification were calculated. The results are shown in Figure 1. The running segment presented a higher correlation (0.82), followed by cycling (0.62) and Lost Time T2 (0.43).

## Discussion

The time lost in T2 showed a correlation of 0.43 with the overall performance of the tri-athletes in competition. This value was even higher than the ones presented by the other two transitions (T1 & T2) and the swimming segment. Losing less time is related to obtaining a better final result. It is a performance factor that should be taken into account when analysing top-level Olympic Triathlon competitions. This new variable varies from 1 to 15 s. It represents a small percentage of a race that lasts slightly less than 2 hours, but it can make a big difference in the final result as the leading positions are often decided by final sprints with differences of a few seconds. Therefore, this time may be a decisive factorregarding the final classification in a triathlon race.

The time lost in T2 is a valid determinant of the final performance of tri-athletes arriving at T2 in the same cycling pack. It depends on two factors: firstly, arriving at T2 in the most advanced position possible within the pack, and secondly, carrying out the necessary actions in T2 as quickly as possible. Some studies tried to identify the changes in speed at decisive points during the competition using a GPS device for each athlete and several video cameras (Vleck at al., 2007). High correlations were found between the speed and position at the start of the swimming (−0.88 for men, −0.97 for women), cycling (0.81 for men, 0.93 for women) and running (−0.94 for men, −0.71 for women). These changes in speed at the beginning and at the end of the segments, together with the transitions, seem to be important factors that may decide the final result. These changes in speed at the start/end of the transitions can be the main reason that could explain the time lost in T1 and T2.

The Olympic Triathlon is a complex sport, not only because three different disciplines are performed back-to-back without stopping the clock, but also because of the speed and precision required during the transitions to pass from one segment to the next (Millet and Vleck, 2000). Transitions are a fundamental part of a triathlon race as they can determine the final results in many competitions. This study takes another step forward in analysing Olympic Triathlon performance as we divided the competition into the following segments: swimming, swimming-cycling transition (T1), time lost in T1, cycling, cycling-running transition (T2), time lost in T2, and running.

The swimming segment showed a low correlation with the final position at the end of the race. This finding is slightly different to the ones obtained in other studies. Landers (2002) analysed 10 international ITU competitions and the correlation of the swimming segment with the overall performance was higher (0.49 versus 0.36). This may be due to the increase in the level of male swimming performance over the last years. It seems the differences in this segment used to be bigger and more decisive in the past than in current competitions. It is very important to be placed in a good position at the end of the swim part, in order to be able to make the first group in the cycling segment (Millet and Veck, 2000). Drafting is also important to consider when covering this segment, in order to save as much energy as possible for the rest of the race (Chatard et al., 1998; Millet et al., 2002). Despite the fact of a low-medium correlation found in the swim, swimming slower does not allow you to compete at the front of the race in further stages of competition. The level of swimming is very high in international elite Olympic Triathlon and a very numerous main pack is formed in the lead whose members present a similar swim speed. This means that the tri-athletes who are not part of the front pack will find it very difficult trying to win the competition.

A low correlation was found between the first transition (T1) and the overall performance. During the cycling segment it is possible to make up the time lost in T1 by catching up with the pack. This could be the reason that would explain the low value found for this correlation. The profiles of most championship routes do not have difficult mountainous sections (steep hills or mountain passes), except for the 2004 Olympic Games, although they do have certain technical difficulties (sharp bends, narrow sections, etc.). Therefore, drafting may be a beneficial tactic in swimming and cycling to increase elite Olympic triathlon performance (Bentley et al., 2007).

The Lost Time in T1 is different for each swimming pack. We identified two packs in our analysis; 1^st^ and 2^nd^ swimming packs when exiting the water. The mean correlations of the 1^st^ and the 2^nd^ swimming pack with the final position at the end of the race were 0.34 and 0.4, respectively. Again, the reason of these medium-low correlations could be the flat routes presented by the cycling sections, where the tri-athletes can make up the time lost in the transition easier.

During the cycling segment in elite triathlon competitions with flat profiles, one or two (three at the most) packs are formed. Normally, those who are not part of the first pack cannot expect to win. This is shown by the medium-high correlation obtained between the cycling segment and the final classification. This result reinforces the hypothesis of the importance of the tactics during this part of the race (Bentley et al., 2007). Significant differences were found in the correlations between the time taken to complete the cycling segment and the overall performance in the different competitions analysed. These differences may be due to two reasons. Firstly, the individual or group tactics adopted by the tri-athletes (aggressive or conservative: trying to break away from the main pack to reach the running segment with a time advantage, or trying to save as much energy as possible to reach the running segment in the best possible condition). And secondly, the orography of the segment (if the profile has mountainous difficulties, the correlation is higher than if the profile is flat). Also, with flat profiles, it is easier and more beneficial to draft in a pack than when riders have to climb mountains, passes or steep slopes (Faria et al., 2005). In this case, the race leads to the creation of smaller packs as was the case in the 2004 Olympic Games. This was the only competition where the correlation between the cycling segment and the final classification was higher (0.86±0.12) than the correlation obtained for the running part (0.76±0.15).

The second transition (cycling-running or T2) has been described as the most important with regard to the final result of the competition (Millet and Veck, 2000). However, we found a low correlation between the time taken for T2 and the final classification. Carrying out a good T2 determines the time lost in T2, which showed a higher correlation with the final result. The running segment has been described as the most decisive segment regarding the performance in triathlon (Slelvert and Rowlands, 1996; Hue et al., 2002; Bentley et al., 2007). In the present study, we obtained the highest correlation with the final classification of all the segments and transitions. This finding reaffirms the data found in the literature. Also, the tactics adopted in the cycling segment will affect the correlation between the running part and the overall performance.

Two different race scenarios that could cause differences were identified. The first one, when the profile of the cycling segment has major orographic difficulties. The 2004 Olympic Games race was the only one that showed a higher correlation for the cycling segment than for the running segment. This was probably due to the fact that the cycling segment was performed over a mountainous profile. The second one, when aggressive tactics leading into breakaways are adopted during the cycling segment. This was the case in the 2006 World Championships, and the correlation between the cycling segment and the overall performance was similar to the one obtained for the running part (0.82 vs. 0.83).

Anthropometry is another factor that may influence performance in the triathlon. The study by Knechtle et al. (2010), related to race time Ironman triathletes anthropometry, found greater relations with the segment of cycling and running, than with swimming. Just as the effects on the recovery phase between competitions, which have been studied in triple ironman triathlon by Knechtle et al. (2009).

According to the competitions analysed, it seems that the tactics adopted by the male tri-athletes during the cycling segment tend to be conservative. Also, it could be that it is more difficult to create circumstances where breakaways reach the running segment with a clear advantage. In addition, the performance level in the cycling segment may be very similar for all the participants, and the fact that there is little collaboration or teamwork may be the reason why breakaways rarely happen. New studies analysing trends during the cycling part in the current format of the World Championship Trial Series competition are needed for further understanding.

Determining the duration of each part of the race (swimming, T1, cycling, T2 & running) was the second aim of the present study. The results show that the average total time found for the men’s Olympic Triathlon competition is similar to the values obtained by other investigations (Landers, 2002). Also, highly significant differences were found for the swimming segment between the present study and the previous ones. Faster swim times were obtained this time, so it seems that the current swim performance is higher nowadays. The average time to complete the cycling segment was similar to the ones reported by other studies. However, the references in the literature analysed events where drafting during cycling was not allowed, so this segment could cause greater fatigue prior to the running segment (Paton and Hopkins, 2005). Finally, the average times for the running segment did not show significant differences.

Comparisons between male winners and all participants were carried out. The results showed highly significant differences for the running time, and significant differences for the total duration of the race (Table 3). As it occurred with absolute times, the running segment showed the greatest difference between the winners and the rest of the participants, indicating that the performance in this segment has a greater impact on the final result. Considering the fact that the swimming/cycling segments offer the possibility of swimming/riding in a pack, and that the level of the participants are very similar, the time differences appear in the last segment. Running in a group has less biomechanical and physiological effects than in the other two segments, and the preceding fatigue has a very significant influence. These findings represent an important difference with the other triathlon modalities where drafting is not allowed during the cycling (e.g. the Ironman). Therefore, the analysis of the competition and final performance factors are different from the Olympic-distance Triathlon competition (Paton and Hopkins, 2005; Bentley et al., 2007).

## Conclusions

Losing less time during T2 has been demonstrated to be related to obtaining a better placing at the end of an Olympic-distance triathlon. Lost Time T2 varies from 1 to 15 s and it represents a small percentage of the race, but it can make a big difference in the final result, as the leading positions are often decided by final sprints with differences of a few seconds.

Competitors need to leave the water in the leading pack to have better chances of winning. The time lost in T1 can be made up in the initial kilometres of the cycling segment, with a medium-low (p<0.05) significance regarding the final placing. The orography of the cycling section and any breakaways can lead to differences in the importance of the time lost in T2. The tactics adopted in the cycling segment may affect the correlation between the running and the final result, which showed the highest values overall.

## Figures and Tables

**Table 1:** Mean (±SD) time for each segment, transition and total time in all the triathlon competitions analysed

**Competition**	**Swim**	**SD**	**CV**	**T1**	**SD**	**CV**	**Bike**	**SD**	**CV**	**T2**	**SD**	**CV**	**Run**	**SD**	**CV**	**Total Time**	**SD**	**CV**
Sydney 2000 O.G	17min59s	24s	2.22	23s	3s	12.81	59min14s	1min27s	2.45	19s	2s	12.70	33min31s	1min57s	5.81	1h51min30s	2min41s	2.42
W.C 2000	18min28s	20s	1.85	46s	3s	7.41	1h1min19s	54s	1.46	34s	16s	20.96	32min57s	1min30s	4.51	1h54min6s	1min57s	1.73
W.C 2001	18min36s	32s	16.95	54s	3s	6.33	58min2s	1min56s	3.33	30s	4s	11.73	34min13s	1min57s	5.63	1h52min16s	3min46s	4.82
W.C 2004	18min30s	21s	10.87	1min7s	4s	6.60	52min19s	1min8s	2.31	37s	3s	7.80	32min35s	1min50s	7.91	1h45min6s	2min37s	3.50
Athens 2004 O.G	18min19s	20s	2.10	18s	1s	7.75	1h3min24s	2min21s	3.71	20s	2s	9.82	34min18s	1min52s	5.46	1h56min20s	3min42s	3.20
W.C 2006	17min51s	25s	2.70	51s	3s	6.46	1h4min13s	2min12s	3.43	35s	3s	9.24	33min32s	1min38s	4.89	1h57min	3min33s	3.04
W.C 2007	17min39s	14s	1.32	41s	4s	10.36	55min38s	1min11s	2.12	21s	3s	15.31	32min19s	1min36s	4.96	1h46min39s	2min22s	2.23
W.C 2008	19min1s	13s	1.16	46s	4s	7.84	59min19s	1min49s	3.10	24s	3s	11.02	34min13s	1min35s	4.65	1h53min43s	2min59s	2.63
Beijing 2008 O.G	18min23s	15s	1.35	28s	2s	6.15	58min52s	21s	0.59	30s	2s	7.60	33min49s	2min5s	6.18	1h52min1s	2min8s	1.91

**Total Time**	**18min19s**	**25s**	**6.89**	**42s**	**16s**	**33.83**	**59min9s**	**3min41s**	**6.73**	**28s**	**7s**	**27.17**	**33min30s**	**44s**	**5.68**	**1h52min5s**	**4min**	**4.58**

Mean in minutes and seconds. SD=in seconds. CV=coefficient of variation in %.

**Table 2:** Comparison of the mean times in each part of the race between all the competitors, top 10 and the winners in all the triathlon races analysed

	**Swim**	**Cycle**	**Run**	**M (95% IC)**	**p**
**Total** (N=538)	1103.2 ± 75.9	3531.1 ± 237.7		−2427.7 (−2450.4 to −2405.1)	0.001
	1103.2 ± 75.9		2007.2 ± 113.9	−904.1 (−926.6 to −881.3)	0.001
		3531.1 ± 237.7	2007.2 ± 113.9	−1523.7 (−1546.4 to −1501.1)	0.001

**Top ten** (N=87)	1088.8 ± 26.8	3490.1 ± 198.2		−2401.1 (−2443.7 to −2358.6)	0.001
	1088.8 ± 26.8		1888.6 ± 49.9	−799.7 (−842.2 to −757.1)	0.001
		3490.1 ± 198.2	1888.6 ± 49.9	−1601.4 (−1644.1 to −1558.9)	0.001

**Winners** (N=9)	1088.8 ± 24.6	3476.1 ± 2.2		−2387.1 (−2528.4 to −2245.7)	0.001
	1088.8 ± 24.6		1862.8 ± 50.5	−774.1 (−915.3 to −632.6)	0.001
		3476.1 ± 2.2	1862.8 ± 50.5	−1613.1 (−1754.4 to −1471.7)	0.001

Values expressed as mean (M) ± SD and 95% CI.p values of analysis of variance comparing differences between groups.

**Table 3:** Comparison of the mean times in each part of the race between all the competitors, top 10, and the winners in all the triathlon races analysed

	**Total (538)**	**TopTen (87)**	**Winners (9)**	**M (95% IC)**	**p**
**Swim**	1103.2 ± 75.9	1088.8 ± 26.8		14.3 (−4.8 to 33.6)	0.186
	1103.2 ± 75.9		1088.8 ± 24.6	14.3 (−41.6 to 70.2)	0.819
		1088.8 ± 26.8	1088.8 ± 24.6	−0.03 (−58.3 to 58.2)	1.000

**Cycle**	3531.1 ± 237.7	3490.1 ± 198.2		40.9 (−22.1 to 104.1)	0.279
	3531.1 ± 237.7		3476.1 ± 200.2	55.1 (−128.4 to 238.4)	0.761
		3490.1 ± 198.2	3476.1 ± 200.2	14.1 (−177.1 to 205.1)	0.984

**Run**	2007.2 ± 113.9	1888.6 ± 49.9		118.6 (89.6 to 147.6)	**0.001**
	2007.2 ± 113.9		1862.8 ± 50.5	144.3 (59.9 to 228.7)	**0.001**
		1888.6 ± 49.9	1862.8 ± 50.5	25.7 (−62.2 to 113.6)	0.771

Values expressed as mean (M) ± SD and 95% CI. p values of analysis of variance comparing differences between groups.
